# Epigenetics in prostate cancer treatment

**DOI:** 10.20517/jtgg.2021.19

**Published:** 2021-08-27

**Authors:** Katelyn Jones, Yanquan Zhang, Yifan Kong, Elia Farah, Ruixin Wang, Chaohao Li, Xinyi Wang, ZhuangZhuang Zhang, Jianlin Wang, Fengyi Mao, Xiaoqi Liu, Jinghui Liu

**Affiliations:** 1 Department of Toxicology and Cancer Biology, University of Kentucky, Lexington, KY 40536, USA.; 2 Department of Biochemistry, Purdue University, West Lafayette, IN 47907, USA.; 3 Markey Cancer Center, University of Kentucky, Lexington, KY 40536, USA.

**Keywords:** Epigenetics, prostate cancer, prostate cancer treatment

## Abstract

Prostate cancer (PCa) is the most commonly diagnosed malignancy among men, and the progression of this disease results in fewer treatment options available to clinical patients. It highlights the vital necessity for discovering novel therapeutic approaches and expanding the current understanding of molecular mechanisms. Epigenetic alternations such as DNA methylation models and histone modifications have been associated as key drivers in the development and advancement of PCa. Several studies have been conducted and demonstrated that targeting these epigenetic enzymes or regulatory proteins has been strongly associated with the regulation of cancer cell growth. Due to the success rate of these therapeutic routes in pre-clinical settings, many drugs have now advanced to clinical testing, where efficacy will be measured. This review will discuss the role of epigenetic modifications in PCa development and its function in the progression of the disease to resistant forms and introduce therapeutic strategies that have demonstrated successful results as PCa treatment.

## INTRODUCTION

Prostate cancer (PCa) continues to be the most common cancer^[1]^ and ranks as the second leading cause of cancer-related death in United States of America males^[1]^. Numerous studies have documented that androgen receptor (AR) signaling continues to participate as a vital component to the development and progression of PCa. AR is a nuclear hormone receptor that becomes activated upon the binding of androgen ligands and dissociates from cytoplasmic chaperone protein HSP90, where it then can self-dimerize and translocate to the nucleus^[2]^. AR has the ability to bind to androgen response elements (ARE), which results in the transcription of target genes and contribution to prostate development and maintenance^[2]^. In early diagnosed stages of the disease, there are successful treatment options to prolong patient survival, such as medical or surgical castration that distribute AR binding, including radical prostatectomy, radiotherapy, or androgen deprivation therapy (ADT). However, PCa can reoccur after these interventions, which is referenced as castration-resistant PCa (CRPC).

CRPC is defined as cancer that continues to proliferate in the absence or depletion of testosterone, compared to early stages that require higher levels of testosterone for survival and formation, but AR signaling remains an essential contributor to PCa progression. This led to the synthesis of Androgen-Signaling Inhibitors (ASI), such as Abiraterone and Enzalutamide (ENZ), which targets androgen synthesis and AR, respectively. Though ASI treatment options have shown impressive results in reducing PCa, enviably, within several months, the disease will progress into terminal ASI-resistant PCa. Currently, there are several various areas of study arising to address the issue of overcoming ASI resistance. Abiraterone acetate is a hormonal treatment for metastatic CRPC (mCRPC) both before and after chemotherapy and significantly reduces androgen production by blocking the enzyme, which illustrates its efficacy^[2]^. ENZ is a second-generation nonsteroidal antiandrogen utilized in the treatment of mCRPC patients and has shown efficacy to provide patients with a reasonable quality of life^[3]^. Though there has been significant success associated with the administration of these treatments, resistance does occur with both Abiraterone and ENZ, possibly from the development of splice variants^[3]^. Wnt signaling has been extensively documented in its involvement in ENZ resistance. The inhibition of both canonical and non-canonical Wnt signaling has demonstrated positive results in re-establishing sensitivity to ENZ treatment in resistant cell lines and tumor models^[3-5]^.

Additionally, we have established that 3-hydroxy-3-methyl-glutaryl-CoA reductase, a crucial enzyme in the mevalonate pathway, is elevated in ENZ-resistant PCa cells, and combination therapy of simvastatin and ENZ could result in significant inhibition of ENZ-resistant cancer cell proliferation in both *in vivo* and *in vitro* models^[6]^. Even in terms of AR splice variants, which play a critical role in the development and progression of CRPC^[7]^. Wang *et al*.^[8]^ demonstrated that treatment with Malat1 small interfering RNA inhibits ARV7 expression in cell lines and significantly reduces tumor growth in ENZ-resistant xenografts. Additionally, Yamamoto *et al*.^[9]^ demonstrated that using antisense oligonucleotides to target both the full-length AR and its splice variants resulted in a suppression of ENZ-resistant cell lines and xenografts proliferation. Due to the increase in understanding the molecular mechanisms that drive PCa, there has been a rising interest in epigenetics related to new therapeutic approaches.

**Figure 1.**
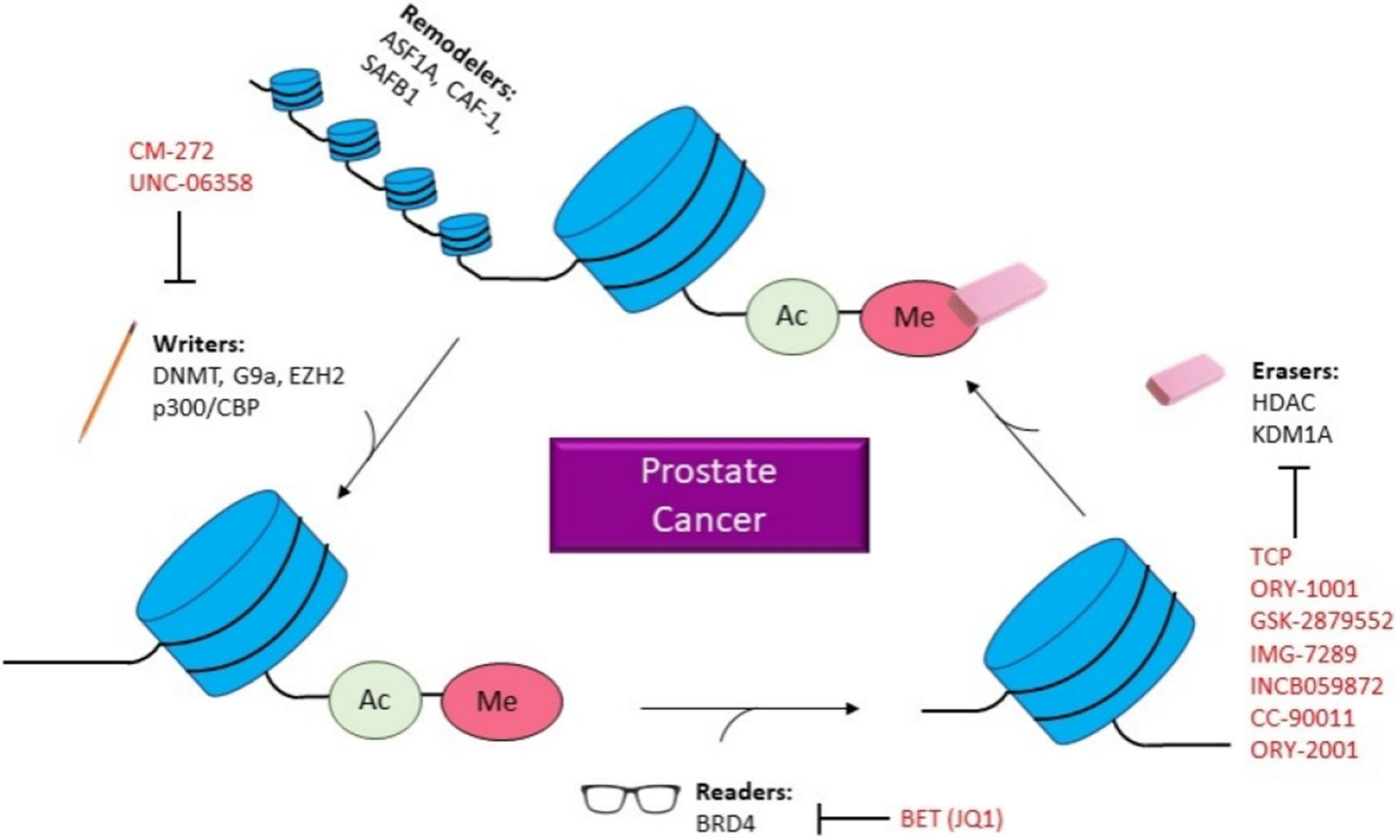
Epigenetics in PCa. Diagram illustrating how epigenetic changes related to prostate cancer and which inhibitors are in current studies. ASF1A: Anti-silencing function 1A hisotne chaperone; CAF-1: chromatin assembly factor 1; SAFB1: scaffold attachment factor B; DNMT: DNA methyltransferase; EZH2: enhancer of zeste 2 polycomb repressive complex 2 subunit; CBP: CREB-binding protein; BRD4: bromodomain containing 4; BET: bromodomain and extra terminal domain; HDAC: histone deacetylases; KDM1A: lysine demethylase 1A; CM-272: 6-methoxy-2-(5-methylfuran-2-yl)-N-(1-methylpiperidin-4-yl)-7-(3-(pyrrolidin-1-yl)propoxy)quinolin-4-amine; UNC-06358: 2-cyclohexyl-6-methoxy-N-(1-propan-2-ylpiperidin-4-yl)-7-(3-pyrrolidin-1-ylpropoxy)quinazolin-4-amine; JQ1: (S)-tert-butyl 2-(4-(4-chlorophenyl)-2,3,9-trimethyl-6H-thieno[3,2-f][1,2,4]triazolo[4,3-a][1,4]diazepin-6-yl)acetate; TCP: tranylcypromine; ORY-1001: idademstat; IMG-7289: bomedemstat HCL; INCB059872: 1-((4-(methoxymethyl)-4-((((1R,2S)-2-phenylcyclopropyl)amino)methyl)piperidin-1-yl)methyl)cyclobutane-1-carboxylic acid compound with 4-methylbenzenesulfonic acid (1:2); CC-90011: besylate; ORY-2001: vafidemstat.

Epigenetics is the study of heritable changes in gene expression, subsequently controlling fate, without affecting actual DNA sequences^[10]^. An abundance of exploration has been accomplished in epigenetics since it was first introduced and defined in the 1950s by Conrad Waddington. These recent discoveries have led to the emergence of operational mechanisms that are composed of three processing steps. These steps can be defined as epigenator, which is a signal that originates from the cellular environment and initiates downstream signaling pathways^[11]^. The epigenetic initiator, which triggers the epigenetic modification at a specific chromatin structure and the epigenetic maintainers that are epigenetic code responsible for ensuring several events such as chemical modifications on DNA or histones molecules, interacts between DNA, RNA, non-coding RNAs, protein, and other chromatin remodeling events^[11]^. Regarding the study of PCa, these aberrant alternations have reinforced the establishment of a context-specific translational profile that favors self-renewal, survival, and invasion and has demonstrated that the accumulation of epigenetic aberrations eventually causes genetic or genomic instability [Figure 1]. Additionally, AR is demonstrated to function in conjunction with various chromatin remodelers and epigenetic players that regulate prostate development and its progression to a malignant phenotype. This review will discuss the essential epigenetic alternations that are critical in comprehending PCa etiology and developments that highlight new biomarkers and therapeutic approaches to PCa.

## EPIGENETIC REGULATORS OF PCA

### Epigenetic writers

Epigenetic codes have commonly been documented to be regulated by writers, readers, and erasers. Writers hold the responsibility to transcribe the epigenetic modifications of DNA and histone proteins^[11]^. These modifications transpire from the addition of various chemical groups utilizing numerous enzymes. An invariable number of modifications have the potential to materialize, but for this review, we will focus on the reactions of methylation and acetylation. DNA and histone proteins are highly prone to methylation, which is the addition of a methyl group to a DNA molecule that may result in a change in the activity of the DNA segment, but will not modify the sequence. Routinely, acetylation is a process in which an acetyl functional group is transferred from molecule to an adjacent molecule and functions by removing the positive charge, thus reducing the N-termini interaction that contains negatively charged phosphates of DNA, exclusively in histones. In this area, we will focus on the addition of these modifications and how they affect the progression and severity of PCa. We will also explore therapeutic methodologies that have been established to address these alterations in function.

#### DNMT and DNA methylation

DNA methylation often plays a role in suppressing gene transcription when located in a gene promoter. DNA methyltransferases (DNMTs) are responsible for transferring methyl groups from the methyl donor S-adenosyl-L-methionine to the 5-position of cytosine residues in DNA, which is critical for mammalian development. The DNMT family has five members, including DNMT1, DNMT2, DNMT3a, DNMT3b, and DNMT3l^[12]^. DNMTs play an important role in genome integrity as their disruption may lead to chromosomal instability and tumor progression^[12,13]^. The main function of DNMT1 is to maintain the methylation status of DNA. As an RNA methyltransferase, DNMT2 usually methylates multiple tRNAs^[14]^. DNMT3a and DNMT3b are reported to contribute the *de novo* DNA methylation.

DNMT3l improves the catalytic activities of DNMT3a and DNMT3b, resulting in the promotion of DNA *de novo* methylation by interacting with DNMT3a and DNMT3b^[15]^. DNA methylation has been shown to play a role in PCa, and DNA methylation marks have been studied for their diagnostic and prognostic values. One of the most recognized DNA methylation events in prostate carcinogenesis is the hypermethylation of the regulatory region of GSTP1, leading to a decrease in gene expression. This hypermethylation event has been found in more than 90% of prostate adenocarcinoma samples and studied for its potential diagnostic and prognostic value^[16]^. Other studies have shown that various genes such as APC, RASSF1a, PTGS2, MDR1, GSTM2, and PENK are hypermethylated in primary and metastatic PCa cells compared to normal prostatic tissues, suggesting that DNA methylation becomes deregulated and may play a role in the prostate carcinogenesis process^[16]^. Both DNA hypermethylation and DNA hypomethylation correlate with prostate carcinogenesis and progression. In a study of 10 normal prostates and 45 prostate tumors, 61 genes were found to be hypermethylated in more than 20% of tumors. A cluster of tumors with hypermethylation of ETV1 and ZNF215 was correlated with ADT resistance in these patients, suggesting a potential use for hypermethylation cluster for prognostic purposes^[17]^. In another study analyzing 84 prostatic tumor tissues with low and intermediate grade PCa, DNA hypermethylation was associated with poorer prognosis and prostate-specific antigen (PSA) recurrence following prostatectomy^[18]^. Comparing DNA methylation in PCa tissues to benign prostatic hyperplasia tissues revealed a higher occurrence of hypermethylation in a group of genes, suggesting a role for these signatures in the diagnostic and prognostic setting of PCa^[18]^. The hypomethylation of MYC’s exon3 is not associated with changes in its expression; however, it was associated with a more aggressive phenotype in the examined cohort^[19]^. In another study and contrary to the established consensus, a group of hypermethylated genes in PCa tumors was associated with increased gene expression^[20]^. In an Iranian study, 35 prostate tumor samples were examined before and after hormone therapy treatment. Treatment with bicalutamide-based drugs for three months induced a significant decrease in the expression of DNMT3A and significant increases in the expression of DNMT3B and two well-established methylated genes, *GSTP1* and *RASSF1*^[21]^. Gravina *et al*.^[22]^ showed that treatment with bicalutamide induced an increase in DNMT activity in PCa that correlated with an increased expression of DNMT3A and DNMT3B. These observations warrant further investigation to understand better the regulation of DNA methylation patterns in PCa, their effects on disease progression, and treatment decisions moving forward.

#### G9a and histone Methylation

G9a, a histone methyltransferase, has the capacity to di-methylate histone 3 at lysine position 9. This epigenetic modification generally represses gene expression^[23]^. However, several studies have demonstrated that G9a also functions as a coactivator of nuclear receptors, such as AR^[24,25]^. Despite the epigenetic function of G9a, the first example of G9A operating as a non-histone lysine methyltransferase, reported that G9A could auto-methylate at the end of its N-terminal^[26]^. Following this trend, CDYL1, WIZ, and ACINUS were discovered as G9A substrates via peptide arrays^[27]^, suggesting that G9A mediated lysine methylation is critical for both histone and non-histone proteins. It has been observed that G9a is overexpressed in a number of cancers^[28,29]^, and elevated G9A protein and its enzymatic activities have been determined under hypoxia stress. For example, Reptin and Pontin, two chromatin remodeling factors, can be methylated by G9A through hypoxia-dependent manners^[30,31]^. Furthermore, methylated Retin contributes to tumor growth and invasive activities via negative regulation of HIF1. Additionally, hypoxia-induced Pontin methylation enhances the ability of proliferation and invasion in breast cancer cells. An alternative study showed that hypoxia-mediated G9A also suppressed RUNX, a tumor suppressor, through histone modification. In addition, hypoxia-mediated G9A amplification decreases apoptosis and increases immature stem-like cancer cells^[32]^. In contrast, hypoxia-mediated G9A represses cell adhesion molecules and contributes to breast tumor motility^[33]^. Additionally, G9A can also promote breast cancer cell survival through driving hypoxia-mediated gene expression. These impacts on cell malignant behavior potentially are caused by FIH-mediated G9A/GLP hydroxylation^[34]^. In addition, G9a also has been found to contribute to the aberrant metabolism of cancer cells. Increased G9a can epigenetically activate the serine biosynthesis, which in turn promotes cancer cell proliferation and survival^[35]^. Also, Fructose-1,6-bisphosphatase (FBP1), a rate-limiting enzyme, can catalyze F-1,6-BP into fructose 6-phosphate in gluconeogenesis. This process can be repressed by G9A mediated epigenetic modification in breast cancer cell lines. Repressed FBP1 contributes to epithelial-mesenchymal transition transformation, promoting cancer cell metastasis^[36]^. Loss of G9A initiates HEPH expression that converts Fe^2+^ into Fe^3+^. Excessive Fe^3+^ will initiate cell cycle arrest machinery^[37]^. Though, there is a lack of evidence that suggests that dysregulation of G9a affects PCa. The interaction between G9a and NKX3.1 contributes to prostate differentiation^[38]^. In addition, G9a plays as a coactivator for PSA induction^[23]^. It suggests that misregulation of G9a may possibly contribute to the generation and progression of PCa. In conclusion, inhibition of G9a may enhance cancer treatment, making it a promising target. The inhibition of G9a has been studied in various cancer types. CM-272, an inhibitor for both G9a and DNMTs, activates immune-related pathway and increase the efficacy of anti-PD-1 immunotherapy [Table 1]^[39]^. Inhibition of G9a with UNC-0638 re-sensitizes pancreatic ductal adenocarcinoma tumors to MEK inhibition and reduces drug-tolerant cells in several cancer cell lines [Table 1]^[40]^.

#### EZH2 and histone methylation

Enhancer of zeste homolog 2 (EZH2) is the essential subunit of the polycomb repressor complex2 (PRC2) and acts as a histone methyltransferase to catalyze tri-methylation of Lys27 on histone H3 (H3K27me3). EZH2 is commonly known to promote the progression of diverse human cancers by H3K27me3-mediated silencing of tumor suppressors^[41,42]^. However, EZH2 can also methylate target genes directly, such as *STAT3*, *GATA4*, and *Jarid2*, to modulate their expression and contribute to cancer development^[43-45]^. In addition to the catalytic function of EZH2 in epigenetic modification, a novel PRC2-independent role of EZH2 as a transcriptional activator was identified by several studies, including NOTCH1, NF-κB, and Wnt signaling^[46-48]^. In the development of PCa, specifically CRPC, EZH2 has been identified to function as a transcriptional coactivator interacting with AR. This functional transfer from a transcriptional suppressor to an activator is driven by the AKT-dependent phosphorylation of EZH2 at Serine-21^[49]^. Recently, it was documented that EZH2 can activate AR signaling via direct binding at the AR promoter region^[50]^. According to these established molecular mechanisms contributing to ADT-resistance acquisition, our lab has questioned whether EZH2 contributes to the resistance of ENZ in CRPC. Our lab determined that EZH2 can bind to the promoter of PSA, resulting in the suppression of its transcription, concluding that pharmaceutical inhibition of EZH2 can overcome ENZ-resistance in CRPC^[51]^. Our findings suggest that the inhibition of EZH2 via existing FDA-approved EZH2 inhibitors can increase the efficacy of ENZ treatment, providing terminal CRPC patients with a novel therapeutic strategy. In addition, we also illustrated EZH2 inhibition could enhance the anti-neoplastic activity of metformin in PCa by reducing the binding of AR to the miR-26a-5p promoter^[52]^. Collectively, these findings suggest that EZH2 could be an effective therapeutic target for PCa, particularly for AR-positive CRPC.

#### p300/CBP and histone acetylation

Histone acetyltransferase p300 and its highly homologous CREB-binding protein (CBP) attach acetyl groups to proteins, including histones, in which DNA is wrapped^[53,54]^. Histone acetylation is a critical method that governs chromatin. When histones are acetylated, chromatin structures in that region will gain a loose conformation, and gene transcription will be promoted^[54]^. It has been reported that p300 and CBP were implicated in the progression of PCa and that deletion of p300 in mice limited PCa progression and extended mice survival^[55]^. The oncogenic roles of p300/CBP in the progression of PCa are usually related to the regulation of AR, the key driver of PCa. p300 can directly acetylate AR, or bind with AR, to enhance its transcriptional activity, consequently inducing oncogenes expression and promoting tumor growth^[55-57]^. In addition to enhancing AR transcriptional activity, p300 can also regulate AR protein level by preventing its degradation^[55]^. These findings highlight p300 as a compelling target for PCa treatment. Indeed, studies have shown that targeting p300/CBP inhibited both androgen-sensitive and CRPC cell growth^[53,57,58]^. In addition, our lab has recently reported a novel mechanism underlying p300 involvement in PCa progression by upregulating PD-L1 expression, thus creating an immune cell-free microenvironment for tumor progression. We found that p300 was recruited to the promoter of CD274 (encoding PD-L1) by transcription factor IRF-1 and resulted in acetylation of histone H3 at the CD274 promoter, and subsequently CD274 transcription. The p300/CBP inhibitor blocked the transcription of CD274 and hindered exosomal PD-L1 secretion. Cutting off PD-L1 secretion at transcription by inhibiting p300/CBP in combination with anti-PD-L1 antibodies demonstrated increased efficacy in a syngeneic mouse model of PCa^[59]^. Our discovery suggests that p300 is not only a modifier but also a co-driver for PCa progression, confirming that p300 could be a compelling target for PCa treatment.

### Epigenetic readers

The framework of modifications constructed by epigenetic writers requires other cellular proteins to both recognize and mediate their effects. Epigenetic readers are protein domains that can bind to these modifications that may be present on DNA and histones. This section will focus on the domains that can be both and recognize methylation and acetylation and therapeutic approaches in PCa.

#### Readers of DNA methylation

DNA methylation is a major epigenetic process that regulates chromatin structure which causes transcriptional activation or repression of genes. The process of DNA methylation is the addition of methyl groups to the correct bases located on the genome by “writer” molecules, known as DNA methyltransferases^[17]^. DNA methylation can provide two different functions. The first function is that DNA methylation directly inhibits transcription factor binding at the gene regulatory region, resulting in transcriptional repression. An alternative operational route is to recruit reader molecules, commonly referred to as methyl-binding proteins (MBP), at the methylated site, which can then attract various members of the chromatin remodeling complex, which will result in transcriptional activation or repression with a dependence on the cellular content. DNA methylation has long been suspected of playing a role in tumorigenesis and cancer progression in various tissue types. Due to this linkage, several drugs have been approved by the FDA, such as Vidaza and Dacogen, which act as DNA methylation inhibitors and are utilized as cancer therapies. These inhibitors operate by reversing the hypermethylated state at the promoter regions of tumor suppressor genes and induce activation of premetastatic genes. In prostate cancer, it has been reported that the knockdown of methyl-binding protein 1 (MBP-1), which functions as a general transcriptional repressor in human PCa cells, results in a delay of cell cycle progression via the inhibition of cyclin A and cyclin B1 expression^[60]^. Additionally, it has been shown that the carboxyl-terminal repressor domain of MBP-1 (MBP-CR) is sufficient for regression of prostate tumor growth in nude mice and suggests that MBP-CR expression has an anti-proliferative effect in human prostate cancer cells compared to the full-length MBP-1 in preventing tumor growth^[60]^.

#### BRD4

The bromodomain-containing family proteins recognize and bind to acetylated lysine residue modifications of histones or proteins, an important class of acetylation readers. The bromodomain was first reported as an evolutionarily conserved domain in proteins of humans, flies, and yeast in 1992^[61]^. It has approximately 110 amino acids and consists of four α helices forming a hydrophobic cavity that identifies acetyl-lysine. There are 42 bromodomain-containing proteins with 61 unique bromodomains In humans, in which differences of the amino acid residues at the acetyl-lysine binding site determine the specificity of binding^[62]^. The BET (bromodomain and extra terminal domain) subfamily proteins have two conserved amino-terminal bromodomains (BD1 and BD2) that are pivotal for recognizing acetylated lysine residues of histones and other non-histone proteins, playing an important role in regulating transcription by recruiting RNA polymerase II (POL II)^[63]^. BRD4 is one of the well-studied BET family proteins that recognize either histone tail or non-histone acetylated modifications at lysine residues. BRD4 was first described as a MED1-interacting protein and occupies thousands of enhancers and promoters related to gene activation^[64]^. BRD4 also works as a critical regulator of the positive transcriptional elongation factor b (P-TEFb) complex via recruiting it to the chromatin and mediates the activation of P-TEFb, consequently phosphorylating and activating RNAPOL II. It is reported that the interaction of BRD4/P-TEFb is crucial for rapid transcriptional reinitiating after mitosis^[65,66]^. Besides recognizing histone acetylation, BRD4 also identifies and binds to the acetylated lysine residues of non-histone. Shi *et al*.^[67,68]^ discovered that BRD4 identifies Tip60-diacetylated of Twist and thereby constructing an activated Twist/BRD4/P-TEFb/RNA-Pol II complex at the WNT5A promoter and enhancer in breast cancer. BRD4 also functions as an atypical kinase to directly phosphorylate Serine 2 of the CTD of RNA POL II, implicating BRD4 as a regulator of transcription^[69]^. Recently, BRD4 also phosphorylates c-MYC at Thr58, resulting in MYC ubiquitination and degradation, suggesting BRD4 negatively regulates MYC level^[70]^. Overall, BRD4 possesses a pivotal role in the regulation of transcription and protein stabilization.

BRD4 plays an oncogenic role and is a potential target of therapy in various cancers. In CRPC, Pawar *et al*.^[71]^ unrevealed that BRD4 physically interacts with AR, and the inhibition of BRD4 disrupts AR recruitment to target gene loci and abrogates AR-mediated gene transcription, including induction of the *TMPRSS2-ERG* gene fusion and its oncogenic activity. The study provides a novel epigenetic approach for the concerted blockade of oncogenic drivers in advanced PCa. In addition, in ER+ breast cancer, Nagarajan *et al*.^[72]^ discovered that BRD4 occupies distal EREs enriched for the histone H3 lysine 27acetyl (H3K27ac) mark and regulates enhancer RNA synthesis by affecting RNAPII recruitment and elongation. Consistently, BRD4 activity is required for the proliferation of ER+ breast and endometrial cancer cells and uterine growth in mice. In conclusion, several studies are focusing on BRD4 as a target for therapy. To inhibit the function of BRD4, a number of selective small-molecules have been developed, which function by blocking the binding of BRD4 to targeted genes via competing for the acetyl-binding pockets^[73,74]^. One of the most popular inhibitors is JQ1, a thieno diazepine-based small molecule, which shows excellent inhibition against the BET subfamily in the low nanomolar range, and is especially effective against BRD4^[74]^. Currently, at least 10 BET inhibitors (BETis) have participated in clinical trials [Table 1]^[75-80]^. It is well reported that PCa-associated *SPOP* mutations cause resistance to BETis via BRD4 accumulation^[77]^. In this regard, besides small-molecule inhibitors, a serial of proteolysis targeting chimera (PROTAC) has recently been developed to target BET proteins for degradation^[78,79]^. Pawar *et al*.^[71]^ found that PROTAC-BETd (ZBC260) effectively induces BRD4 degradation and results in BETi-resistant cells revers into sensitive cells to BETis. It suggests that the utilization of both small molecule inhibitors and PROTACs makes targeted therapy of BRD4 an effective therapy in various cancer models.

Currently, there is a lack of BETis, including JQ1 approved by the FDA for clinic application due to dose-limiting toxicity. Given that combination treatment is a classic strategy to reduce the monotherapy dosage, Mao *et al*.^[80]^ proposed that the PLK1 inhibitor GSK461364A could synergistically combine with BRD4 inhibitor JQ1 in the treatment of CRPC. The co-inhibition of BRD4 and PLK1 resulted in delayed cell growth, substantial cell apoptosis, and catastrophic cell cycle arrest in aggressive human CRPC cells. The significant improvement of efficacy in combining a PLK1 inhibitor and BRD4 inhibitor suggests a novel therapy for clinical trials.

### Epigenetic erasers

Though epigenetic markers in post-translational modifications on histones are covalently linked to DNA, they are not permanently bound to the structure. Epigenetic erasers are a group of enzymes that maintain the ability to oppose the activity of writers and catalyze the removal of epigenetic alternations. This removal relieves its effect on transcription, resulting in the modulation of gene expression^[17]^. In the section, we emphasize the enzyme responsible for removing methyl and acetyl groups while discussing its role in the prostate and introducing therapeutic tactics.

#### HDAC

In contrast to histone acetyltransferase transferring acetyl group to histones, histone deacetylases (HDACs) remove acetyl groups from histones, resulting in a more condensed form of chromatin and gene silencing. To date, four HDAC classes have been identified in humans^[81-83]^. Class I HDACs, consisting of HDACs 1, 2, 3, and 8, are mainly localized in the nucleus and expressed in most tissues. Class II, consists of HDACs 4, 5, 6, 7, 9, and 10, are localized both in the nucleus and the cytoplasm. Class III HDACs are homologs of yeast silent information regulator 2 and consist of SIRT 1-7. Class IV HDAC consists of HDAC 11. Class I, II, and IV HDACs have a zinc coordinated active site, whereas Class III HDACs are dependent on coenzyme nicotinamide adenine dinucleotide for deacetylase activity.

#### HDAC role in PCa and therapeutic approaches

In cancer cells, high expression of HDACs results in the deacetylation of histone proteins, which causes DNA to be wrapped tightly by histones, thereby inhibiting gene expression. If the affected genes are tumor suppressors, the neoplastic proliferation of cells and cancer may result^[83]^. It has been reported that Class I HDACs (HDAC 1, 2, and 3) are highly expressed in PCa, specifically in CRPC^[84,85]^. Additionally, evidence has shown HDACs play a positive role in regulating the AR protein level and its transcriptional activity^[86-88]^. Therefore, it seems that HDACs could exhibit opposing pro- and anti-tumorigenic roles in PCa cells. In addition, HDAC inhibition could induce cell cycle arrest, apoptosis, autophagy, and reactive oxygen species generation^[82,89]^. The support from these discoveries has led to the initiation of several clinical trials of HDACs inhibitors in PCa treatment, including vorinostat, pracinostat, panobinostat, and romidepsin. However, none were recommended to continue phase III trials due to either toxicity or disease progression^[82]^. In summary, the function of HDAC in PCa and whether HDAC could be an effective target in the treatment of PCa is still ambiguous and requires further investigation to reach a conclusion.

#### Demethylase of histones

Histone lysine demethylases (KDMs) are a class of enzymes that can remove methyl groups from nucleic acids, proteins, and specifically histones. The first human KDM was reported in 2004^[90]^. To date, several lysine-specific demethylase isoforms were discovered and characterized. Since their discovery, KDMs have been found to be deregulated in various cancers, such as non-small cell lung, breast, colorectal, pancreatic, *etc*.^[87,91]^. In PCa, KDMs may act as either tumor suppressors or oncogenes, which is dependent on the genes regulated by the KDMs.

Recently, Gao *et al*.^[92]^ found that KDM1A is demethylation of FOXA1 at K270, and methylation of this residue decreases FOXA1 stability and activity. Inhibition of KDM1A, therefore, induces FOXA1 instability and results in FOXA1 chromatin dissociation, thus leading to loss of AR transcriptional activity. Consistent with this finding are several previously completed studies^[93-95]^, which have demonstrated that KDM1A is required for the AR transcriptional activity regulation, both AR and AR variants, confirming its involvement in the progression of PCa. Interestingly, a recent study^[96]^ showed that KDM1A could promote the survival of PCa cells independently of its demethylase function. This effect is explained by the activation of a lethal PCa gene network in collaboration with KDM1A’s binding protein, ZNF217. Numerous KDM1A inhibitors, such as TCP, ORY-1001, GSK-2879552, IMG-7289, INCB059872, CC-90011, and ORY-2001, have been reported and are presently being investigated in clinical trials for cancer treatment [Table 1]^[97]^. Several have exhibited significantly improved potency and selectivity. In addition to KDM1A, KDM3A^[94]^, KDM4A/4B^[98-101]^, and KDM6A/6B^[102]^ were also identified as coactivators of AR and play critical roles in PCa progression, thus characterizing them as potential therapeutic targets. These findings highlight the roles of KDMs in PCa initiation and progression, suggesting that targeting KDMs’ activity may provide a new strategy for PCa treatment.

### Chromatin remodelers

Due to the budding advancements in high-throughput epigenomic approaches, visualizing chromatin structures and how their alternations result in disease development and progression has become an increased area of study^[103]^. Chromatin remodeling can be defined as the rearrangement of chromatin from a condensed state to a transcriptionally accessible state^[104]^. This rearrangement allows for transcription factors or DNA binding proteins to access DNA and control gene expression. This section will focus on chromatin remodeling as a compelling target for PCa therapeutic approaches.

#### ASF1A

Anti-silencing function 1A histone chaperone (ASF1A) is a major isoform of ASF1, a small histone chaperone of the H3/H4 family and conserved from yeast and human cells^[105]^. As the major isoform of ASF1 in human cells, ASF1A is ubiquitously expressed in all tissues and throughout the cell cycle^[106]^. The elevated expression of ASF1A positively correlates with the level of H3K56Ac^[107]^, which is a mark of newly replicated chromatin as well as replication-independent histone replacement. ASF1A contributes to the resistance of DNA damage tolerance because of its ability to promote double-strand break (DSB) repair by non-homologous end joining. ASF1A deficiency and loss will render cells more sensitive to DSBs. For example, knockout ASF1A leads to the introduction of DSBs, which sensitizes cancer cells to radiotherapy, chemotherapy, and immunotherapy^[108,109]^. ASF1A has emerged as an oncogenic driver. Regarding several cancer cases, ASF1A accumulation is a general characteristic that occurs in tumorigenesis^[110]^. ASF1A is highly expressed in prostate cancer cells, and its overexpression is associated with poor prognosis in cancer patients^[104,111-113]^. Some reports have shown that blocking the expression of ASF1A by RNA interference^[108,111]^, small inhibitors, and chemotherapy drugs^[110]^ can effectively inhibit the proliferation and growth of tumors and improve the sensitivity to anti-cancer drugs and immunotherapy^[108,109]^.

#### CAF-1

Histone chaperone chromatin assembly factor-1 (CAF-1) is composed of p150 large unit (CHAF1A), p60 middle unit (CHAF1B), and p48 small unit (RbAp48) and is involved in the deposition of (H3-H4)_2_ tetramer onto DNA^[114]^. During replication, CAF-1 receives (H3-H4)_2_ tetramer from another histone chaperone ASF1A and then deposits the histone onto the newly synthesized daughter DNA strands^[114,115]^. It has been reported that the dysregulation of histone assembly is closely associated with certain human diseases such as cancer^[116]^. Indeed, CAF-1 has been shown to be a marker of proliferating cells^[117]^, and depletion of CAF-1 induces cell death, possibly due to the activation of DNA damage response pathway^[118]^. Specifically, in PCa, the middle unit of CAF-1 is a prognostic marker of adverse outcomes for patients^[111]^, and inhibition of ASF1A suppresses the growth of PCa^[119]^. These interesting results raise the possibility that targeting chromatin assembly in PCa is a potential treatment for PCa patients.

#### SAFB1

SAFB1 (scaffold attachment factor B1) is a nonenzymatic architectural component of the chromatin that was first identified to bind adenine- and thymine-rich scaffold/matrix attachment (S/MAR) regions^[120]^ to divide the genome into 5-200 kb topological domains. SAFB1 was previously assumed to mediate chromatin looping to modulate long-range chromatin interactions and higher-order chromatin structure^[119]^. SAFB1 is a component of the DNA damage response and cooperates with histone acetylation to allow for efficient gH2AX spreading and genotoxic stress signaling. SAFB1 undergoes a highly dynamic exchange at damaged chromatin in a poly (ADP-ribose)-polymerase 1- and poly (ADP-ribose)-dependent manner and is required for unperturbed cell cycle checkpoint activation and guarding cells against replicative stress^[121]^. Meanwhile, SAFB1 regulates RNA polymerase II-dependent transcription of targeted genes^[119]^. There is a potent transcriptional repression domain at the C-terminal region of SAFB1, which mediates the transcriptional repression activity. Particularly, SAFB1 binds to nuclear receptors^[122,123]^ and suppresses immune regulators and apoptotic genes^[124]^. SAFB1 attenuates ER*α* transcriptional activity via its interaction with the ER*α* DNA-binding domain in a ligand-independent manner^[125]^. Low levels of SAFB1 were found to correlate with worse outcomes in breast cancer patients^[126]^. In addition, SAFB1 is also reduced with disease progression in a cohort of human PCa, including metastatic tumors. SAFB1 binds to AR and is phosphorylated by the MST1 (Hippo homolog) serine-threonine kinase, an AR repressor, and MST1 localization to AR-dependent promoters is inhibited by depletion of SAFB1. Meanwhile, SAFB1 interacts with Enhancer of Zeste 2 Polycomb Repressive Complex 2 (EZH2) at ARE of chromatin. Knockdown of SAFB1 in androgen-dependent LNCaP cells results in upregulation of AR and PSA levels, stimulating the growth of cultured cells and subcutaneous xenografts and promoting a more aggressive phenotype, which is consistent with a negative AR regulatory function^[127]^. Collectively, SAFB1 functions as a tumor suppressor in both breast cancer and PCa.

#### Epigenetics and genetic instability

Epigenetics and genetics have been described as separate entities, participating in carcinogenesis via independent mechanisms^[128]^. However, recent publications have unveiled crosstalk that occurs between genome and epigenome factors that could produce novel therapeutic strategies in PCa^[127]^.

Microsatellites are highly polymorphic, short-tandem repeat sequences dispersed throughout the genome^[128]^. The instability of these repeats at multiple loci can result in mismatch repair errors and other genetic issues. Loss of heterozygosity (LOH) has been reported to strongly correlate with increasing malignancy in prostate carcinoma^[129]^. Recently it has been documented that chromosomal instability, including MSI/LOH, has been categorized as a distinct type of genetic instability characteristic in regards to prostate cancer^[130]^. Epigenetic processes such as hypermethylation of tumor suppressors, histone modification, and hypomethylation of oncogenes have been documented to eventually create genetic instability in the forms of MSI, LOH, allelic loss, single nucleotide polymorphisms (SNPs), and chromosomal aberrations. DNA methylation alterations could induce loss of heterozygosity and lead to a progression in prostate cancer^[129]^. It has been reported ten-eleven translocase 2 (TET2), enzyme-mediated DNA demethylation, exhibits high mutation rates (10%-20%) and extensive loss of heterozygosity (~60%) in metastatic prostate tumors. Genome-wide association studies have also shown increased PCa risk linked to an intergenic TET2-proximal SNP (rs7679673)^[131]^. Additionally, Baylin and Jones have reported that cancers with hypermethylated MGMT are susceptible to genetic mutations in critical genes such as *p53* or *KRAS*. *MLH1*, a mismatch repair gene, plays an important role in genomic instability. It has been reported that promoter hypermethylation results in loss of function of this gene and causes MSI in several cancers^[132]^.

Regarding histone modification, deregulated Polycomb Repressor Complex 2 mediated epigenetic modifications have been shown to cause genetic instability, malignancy, and cancer development through abnormal tumor suppressor gene expression, DNA damage response, and DNA replication^[133]^. BRD4 acetylates histone H3 at the K122 residue, and this thereby perturbs a salt bridge, leading to nucleosome instability^[134]^. It has been recently documented that targeting genetic instability with possible PARP could be utilized as a novel therapeutic approach in prostate cancer treatment. Epigenetic changes, such as DNA hyper- and hypomethylation, can cause genetic instability, such as LOH/MSI, in various cancer types. Multiple genetic and epigenetic abnormalities in PCa suggest that co-targeting both epigenetic changes and genetic instability could become a novel therapeutic strategy in PCa treatment.

## CONCLUSION

AR has been a critical target for the treatment of PCa, and while ADT has been effective in preventing cancer cell proliferation, progression to a more aggressive phenotype is inevitable. In this review, we discussed the various epigenetic changes which contribute to the further advancement and progression of PCa via the activation of various oncogenic pathways. We also explored novel therapeutic approaches established by our lab and drug treatment strategies that have demonstrated impactful success. Due to recent discoveries in the understanding of the mechanisms of maintained AR signaling in castration-resistance PCa, targeting these epigenetic changes that facilitate AR target gene activation has a highly possible and promising potential in developing novel therapeutic approaches. Though whether targeting these factors’ stability will produce toxic or ineffective effects is obscure, pre-clinical trial data gathered and documented by our lab indicates that clinical trial participation could result in highly efficient and optional treatment methods.

## Figures and Tables

**Table 1.** Inhibitors for epigenetic changes

Inhibitor	Target	Clinical trial phase
CM-272	G9a	Not in clinical trial
UNC-0638	G9a	Not in clinical trial
TCP	KDM1A	Phase 1/2
ORY-1001	KDM1A	Phase 1
GSK-2879552	KDM1A	Phase 1/2
IMG-7289	KDM1A	Phase 2
INCB059872	KDM1A	Phase 1/2
CC-90011	KDM1A	Phase 1
ORY-2001	KDM1A	Phase 2
BET (JQ1)	BRD4	Phase 1

CM-272: 6-methoxy-2-(5-methylfuran-2-yl)-N-(1-methylpiperidin-4-yl)-7-(3-(pyrrolidin-1-yl)propoxy)quinolin-4-amine; UNC-06358: 2-cyclohexyl-6-methoxy-N-(1-propan-2-ylpiperidin-4-yl)-7-(3-pyrrolidin-1-ylpropoxy)quinazolin-4-amine; BET: bromodomain and extra terminal domain; JQ1: (S)-tert-butyl 2-(4-(4-chlorophenyl)-2,3,9-trimethyl-6H-thieno[3,2-f][1,2,4]triazolo[4,3-a][1,4]diazepin-6-yl)acetate; TCP: tranylcypromine; ORY-1001: idademstat; IMG-7289: bomedemstat HCL; INCB059872: 1-((4-(methoxymethyl)-4-((((1R,2S)-2-phenylcyclopropyl)amino)methyl)piperidin-1-yl)methyl)cyclobutane-1-carboxylic acid compound with 4-methylbenzenesulfonic acid (1:2); CC-90011: besylate; ORY-2001: vafidemstat.
